# Tipping the Balance in the Powerhouse of the Cell to “Protect” Colorectal Cancer

**DOI:** 10.1371/journal.pgen.1002758

**Published:** 2012-06-07

**Authors:** Dawit Kidane, Joann B. Sweasy

**Affiliations:** Departments of Therapeutic Radiology and Genetics, Yale University School of Medicine, New Haven, Connecticut, United States of America; Baylor College of Medicine, United States of America

Mitochondria are the bioenergetic centers of eukaryotic cells that produce ATP through oxidative phosphorylation. A byproduct of oxidative phosphorylation is the generation of reactive oxygen species (ROS). Cancer cells exhibit high basal levels of oxidative stress due to activation of oncogenes, loss of tumor suppressors, and the effects of the tumor microenvironment [1]. A large body of evidence suggests important roles for oxygen free radicals in the mutagenesis that drives carcinogenesis [2], the expansion of tumor clones, and the acquisition of malignant properties [3].

**Figure 1:**
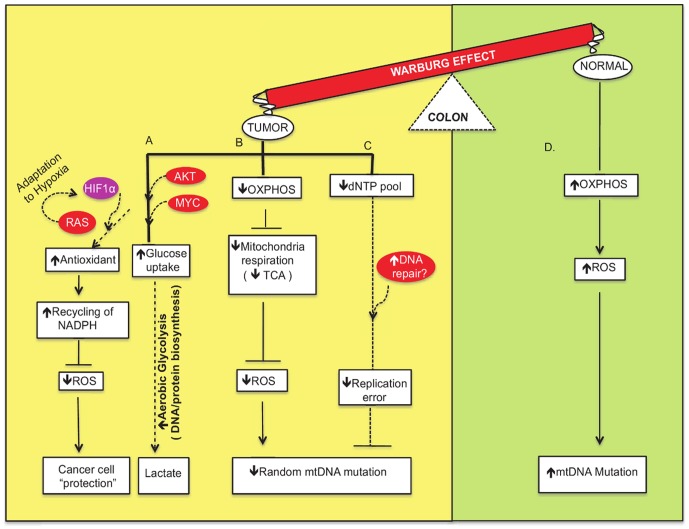
Network modeling of the interconnections among the crucial factors involved in metabolic flow and signaling pathways of the Warburg effect to “protect” the cancer cells. (A) Cancer cells make use of their nutrient-rich environment by taking in glucose and converting it into molecular precursors by aerobic glycolysis (shown in yellow). This is mediated by activation of proto-oncogenes such as AKT and MYC and other genes in important growth factor signaling pathways. Moreover, RAS-mediated activation of HIF1 induces adaptation to hypoxic environments and promotes “niches” that are conducive to cancer cells. In addition, the use of antioxidants and recycling of NADPH as defense mechanisms to sequester ROS favor the survival of cancer cells. (B) Oxidative phosphorylation (OXPHOS) impairment leads to crippled mitochondrial respiration. The dysfunctional TCA cycle generates fewer reactive oxygen species that may or may not induce DNA damage, and this subsequently leads to fewer mitochondrial DNA mutations. (C) Efficient repair of mtDNA leads to fewer mutations and less mitochondrial dysfunction. (D) In contrast, normal, low proliferative cells utilize OXPHOS and generate ROS that induce mtDNA damage and increase mutation frequency (shown in green).

Several studies have reported that a high frequency of clonal and, therefore, selected mitochondrial mutations are present in a variety of human tumors [4]–[15]. Mitochondrial DNA (mtDNA) mutations in cancer cells include intragenic deletions, missense and chain-termination point mutations, and alterations of homopolymeric sequences that result in frameshift mutations [16], [17]. The biological impact of a given mutation may vary, depending on the proportion of mutant mtDNAs carried by the cell. The assumption from these studies is that this high frequency of clonal mutations arises from the ROS produced in mitochondria by the escape of oxygen free radicals during oxidative phosphorylation, and that these mutations play a role in driving cancer (Figure 1). Therefore, genomic instability of mitochondria was thought to be a hallmark of cancer.

Cancer arises from a mutator phenotype and it has been established that the random mutation rate of the nuclear DNA of tumors is quite high [18]. However, little is known about the random mutation rates of mitochondria derived from tumors. In this issue of *PLoS Genetics*, Ericson and colleagues [19] report their striking finding that the random mutation frequency of colorectal tumor mtDNA is significantly lower than that of nuclear DNA or of mtDNA in the surrounding normal tissue. These results were obtained using the random mutation capture assay that was developed to measure random, and not clonal, mutations [19]. This group also shows that the mitochondria from the colon tumors use aerobic glycolysis rather than oxidative phosphorylation to generate energy for the cells. Importantly, this metabolic shift from oxidative phosphorylation to aerobic glycolysis is likely to produce fewer ROS that damage mtDNA and lead to mutagenesis, mitochondrial dysfunction, and cell death.

Seven decades ago, Warburg discovered that mitochondria in cancer cells metabolize glucose by aerobic glycolysis and suggested that this was a result of impaired mitochondrial function that contributes to tumorigenesis [20], [21]. Recently, Thompson and colleagues suggested that mitochondrial function itself is not impaired in cancer cells that metabolize glucose by aerobic glycolysis, and that this type of anabolic metabolism is critical for the production of essential cellular building blocks including dNTPs, amino acids, and lipids [22] (Figure 1). Growth factor signaling by activated AKT, Myc, and other proto-oncogenes results in altered mitochondrial metabolism [22]. For example, a majority of human tumors harbor mutations in the AKT gene, and activated AKT enhances glucose uptake, allowing cells to maintain a higher than adequate level of ATP [23]. What is unknown is whether the colon tumors that were characterized by the Bielas group [19] harbor activated proto-oncogenes. Nevertheless, the picture that now emerges is that aerobic glycolysis enhances growth of cancer cells by anabolic metabolism without producing high levels of ROS that could inactivate the power supply of the cell.

Other explanations for the low frequency of random mitochondrial mutations in colon tumors include highly efficient DNA repair (for excellent review see [24]) and the coupling of antioxidant proteins to the NADPH/NADP balance (Figure 1). Recent studies have demonstrated that mtDNA is repaired by a variety of mechanisms, including short- and long-patch base excision repair, mismatch repair, and homologous recombination [24]–[27]. The sanitation of dNTPs is also likely to result in fewer mutations. Antioxidant proteins may also play a role in the low random mutation rate observed in mitochondria. Proteins such as reduced glutathione (GSH) or thioredoxin that are closely coupled to the NADPH/NADP balance can inactivate ROS [28], [29]. The presence of NADPH and other free radical scavengers may be more pronounced in the colorectal tumor environment to neutralize the impact of oxidative stress (Figure 1).

What are the implications of the findings of Ericson et al. [19] for future cancer therapy strategy or biomarker discovery? Cancer cells exhibit increased uptake of glucose, which increases the bioenergetic competence required of malignant cells. This metabolic feature has led to the hypothesis that inhibition of glycolysis may abolish ATP and important precursor generation in cancer cells and thus may preferentially kill the malignant cells [30], [31]. Nuclear genetic and epigenetic changes have been the cornerstone of such studies. In addition to these strategies, it is likely that mutated genes that function to alter mitochondrial metabolic competence in tumors, including *HIF-1* and *MYC*, will emerge as new drug targets and molecular markers of prognosis and responses to therapy. In addition, targeting mtDNA repair proteins could serve as a potential alternative approach to kill cancer cells. The work of the Ericson et al. [19] adds significantly to our understanding of the roles of mitochondria in supporting the growth of tumors. In combination with recent findings regarding mitochondrial metabolism in cancer cells, this groundbreaking finding suggests that novel drugs that target the powerhouse of cancer cells are likely to make a significant impact on cancer treatment.
